# Unlocking the potential of glyphosate-resistant bacterial strains in biodegradation and maize growth

**DOI:** 10.3389/fmicb.2023.1285566

**Published:** 2023-12-20

**Authors:** Waqas Mohy-Ud-Din, Feng Chen, Safdar Bashir, Muhammad Javed Akhtar, Hafiz Naeem Asghar, Zia Ur Rahman Farooqi, Usman Zulfiqar, Fasih Ullah Haider, Aneeqa Afzal, Mashael Daghash Alqahtani

**Affiliations:** ^1^Institute of Soil and Environmental Sciences, University of Agriculture, Faisalabad, Pakistan; ^2^Department of Soil and Environmental Sciences, Ghazi University, Dera Ghazi Khan, Pakistan; ^3^Institute of Marine and Environmental Technology, University of Maryland Center for Environmental Science, Baltimore, MD, United States; ^4^Department of Agronomy, The Islamia University of Bahawalpur, Bahawalpur, Pakistan; ^5^Key Laboratory of Vegetation Restoration and Management of Degraded Ecosystems, South China Botanical Garden, Chinese Academy of Sciences, Guangzhou, China; ^6^Department of Chemistry, University of Agriculture, Faisalabad, Pakistan; ^7^Department of Biology, College of Science, Princess Nourah bint Abdulrahman University, P.O. Box 84428, Riyadh 11671, Saudi Arabia

**Keywords:** biodegradation, contamination, herbicides, organophosphates, rhizobacteria

## Abstract

Glyphosate [N-(phosphonomethyl)-glycine] is a non-selective herbicide with a broad spectrum activity that is commonly used to control perennial vegetation in agricultural fields. The widespread utilization of glyphosate in agriculture leads to soil, water, and food crop contamination, resulting in human and environmental health consequences. Therefore, it is imperative to devise techniques for enhancing the degradation of glyphosate in soil. Rhizobacteria play a crucial role in degrading organic contaminants. Limited work has been done on exploring the capabilities of indigenously existing glyphosate-degrading rhizobacteria in Pakistani soils. This research attempts to discover whether native bacteria have the glyphosate-degrading ability for a sustainable solution to glyphosate contamination. Therefore, this study explored the potential of 11 native strains isolated from the soil with repeated glyphosate application history and showed resistance against glyphosate at higher concentrations (200 mg kg^−1^). Five out of eleven strains outperformed in glyphosate degradation and plant growth promotion. High-pressure liquid chromatography showed that, on average, these five strains degraded 98% glyphosate. In addition, these strains promote maize seed germination index and shoot and root fresh biomass up to 73 and 91%, respectively. Furthermore, inoculation gave an average increase of acid phosphatase (57.97%), alkaline phosphatase (1.76-fold), and dehydrogenase activity (1.75-fold) in glyphosate-contaminated soil. The findings indicated the importance of using indigenous rhizobacteria to degrade glyphosate. Therefore, by maintaining soil health, indigenous soil biodiversity can work effectively for the bioremediation of contaminated soils and sustainable crop production in a world facing food security.

## 1 Introduction

Glyphosate (GP) is the major component in a broad-spectrum herbicide formulation used in almost all agricultural countries to eradicate weed infestation. The accessibility of resistant crops against GP supports the frequent application of herbicides without sacrificing crop yields (Vats, 2015). Glyphosate inexorably enters the rhizosphere by direct spraying, being washed off from the surface of leaves, or traveling down through the plants to root exudates after application (Kryuchkova et al., 2014). Different complexes are formed in the soil after receiving the GP molecule and are adsorbed on humus particles (Young, 2013). Application of phosphorous (P) fertilizers produces ions of the phosphate group, which contend with GP for binding sites on clay particles. Phosphate ions take the front in attachment. Glyphosate residue persists in soil and affects the microbial communities as well, as it is also available for roots to uptake in the plant's body (Achary et al., 2017). After applying GP, it directly acts by hindering the biosynthesis of aromatic compounds but also reduces the transport of microelements such as manganese (Mn), zinc (Zn), iron (Fe), and calcium (Ca) (Kanissery et al., 2019), which is essential for chlorophyll production (Kryuchkova et al., 2014). Residues of glyphosate and amino methyl phosphonic acid (AMPA) in food consumed by humans are potentially toxicologically concerning if they exceed acceptable daily intake levels. Traces of GP and amino methylphosphonic acid (AMPA) have been detected in plant and animal tissue, indicating that residues exist in various food sources (Bai and Ogbourne, 2016). Hence, despite the low concentrations of GP residues that endure over time, their extensive usage may lead to their accumulation, thereby posing a potential threat to the health of animals and humans. This is due to their prolonged exposure to residues in the food and water they ingest. It has been confirmed that GP is present in the organs and urine of numerous farm animals and farmers. Furthermore, it was observed that residues were present in the urine of 60–80% of the overall population in the United States, with median and maximal concentrations of 2–3 and 233 μg L^−1^, respectively. According to a study conducted by Costas-Ferreira et al. (2022), it was discovered that 44% of the European population had residues present in their urine. However, these residues' average and maximum concentrations were <1 μg L^−1^ and 5 μg L^−1^, respectively. According to existing literature, GP has been identified as one of the carcinogenic substances capable of generating organ failure. This is achieved by the inhibition of acetylcholinesterase and the induction of oxidative stress in non-mammalian species. Amino methylphosphonic acid serves as a crucial metabolite of GP, commonly detected in soil, surface water, and groundwater. Several *in vitro* toxicity investigations have revealed that AMPA exerts an impact on erythrocytes in humans and has the potential to induce chromosomal abnormalities in fish (De Brito Rodrigues et al., 2019).

Glyphosate residue in the rhizosphere changes the biomass of microorganisms in the soil and stimulates the growth of phytopathogenic fungi such as *Fusarium* and *Phytophthora* (Glick and Gamalero, 2021). Glyphosate also reduces the abundance of beneficial rhizospheric communities, such as Mn-reducing bacteria and indole acetic acid-producing rhizobacteria. It has also been observed that GP has a harmful impact on bacteria that transform Mn and indole acetic acid production. In *Euglena* species, glyphosate causes a 20% decrease in respiration and photosynthetic rates (Singh et al., 2020a).

Cereals are one of the essential food crops all over the globe. Maize (*Zea mays* L.) is a high-value crop among cereals. It is one of the highly consumed staple foods all over the world. It is used in the food, paper, and biofuel energy production industries, and its vegetative part is utilized as livestock feed (Jacobsen et al., 2013). Due to its different purposes, its need is rising day by day. There is a dire need to meet up with the growing demand constantly. Still, its yield production is hindered by weed infestation (Arruda et al., 2013). Farmers have applied excessive doses of GP-based herbicide to reduce weed infestation. Several applications of GP in the same crop field gradually decrease the degradation potential of native biota due to its residual toxicity (Guo et al., 2022).

In soils, most of the GP is degraded by microbial communities (Mohy-Ud-Din et al., 2024). The GP degradation rate depends on soil characteristics, climatic circumstances, and its availability to microorganisms (Guijarro et al., 2018). The major by-product of GP is AMPA. The GP and AMPA molecules are characterized by a strong carbon and phosphorous bond, which shows resilience to physiochemical factors. Still, enzyme produce by microbes breakdown the compound (Singh et al., 2020a). With the development of environmentally friendly and cheap remediation techniques, bioremediation is a promising approach to decontaminating the soil using GP-degrading bacteria. Until now, microbial communities that can degrade GP as nutrients, i.e., phosphorous (P), carbon (C), and nitrogen (N) sources, during metabolism or co-metabolism have been characterized and identified (Elarabi et al., 2020). Even though a variety of organophosphate-degrading bacteria have been reported, there still has been limited literature that shows bacteria-mediated techniques for removing GP-contaminated soils at higher concentrations. Ermakova et al. (2010) stated that inoculation of GP-tolerant bacterial strains into contaminated soil considerably increases the rate of GP degradation.

The weak argument for inoculation of GP-tolerant bacterial culture alone is that bacteria must depend on themselves to ensure a niche through which they can acclimatize, compete with the native microbial communities, and have an adequate quantity of food source. This kind of problem can be solved by using bacteria with plant association. In this situation, bacteria form a biofilm with roots and survive under stress by utilizing root exudates as a supplementary source of nutrients, which may significantly strengthen the biodegradation process. In turn, PGPR exerts favorable impacts on plants beyond their capability for auxin production, solubilization of phosphorous, and nitrogen fixation (Gouda et al., 2018). In addition, GP-tolerant bacteria may keep the plant roots resistant to the colonization of phytopathogenic fungi. Given the extensive use of GP, even the minor effects on soil communities reported earlier warrant further work. Several experimental studies have evaluated the effects of GP on soil communities from both agricultural (Weaver et al., 2007) and forest soils (Ratcliff et al., 2006), as well as rhizospheric soil (Kremer and Means, 2009).

Maize is one of the fastest-growing crops and quick response to amendments. Its fast-growing nature and higher biomass production under favorable conditions could favor the biodegradation of GP residue in the soil. Moreover, there is a need to explore the indigenously existing GP-degrading bacteria in Pakistani soils, which should be explored. The hypothesis posited that applying PGPR, which is plant growth-promoting rhizobacteria indigenous to the area, can enhance plant growth and facilitate the degradation of GP residues. This study aimed to examine the impact of rhizobacteria that degrade GP and mitigate GP phytotoxicity. Additionally, the study sought to elucidate the resulting morphological and physiological traits of maize plants and enhance soil enzymatic activities in soil that was spiked under ambient conditions.

## 2 Methodology

### 2.1 Soil sample sites

**Figure 1:**
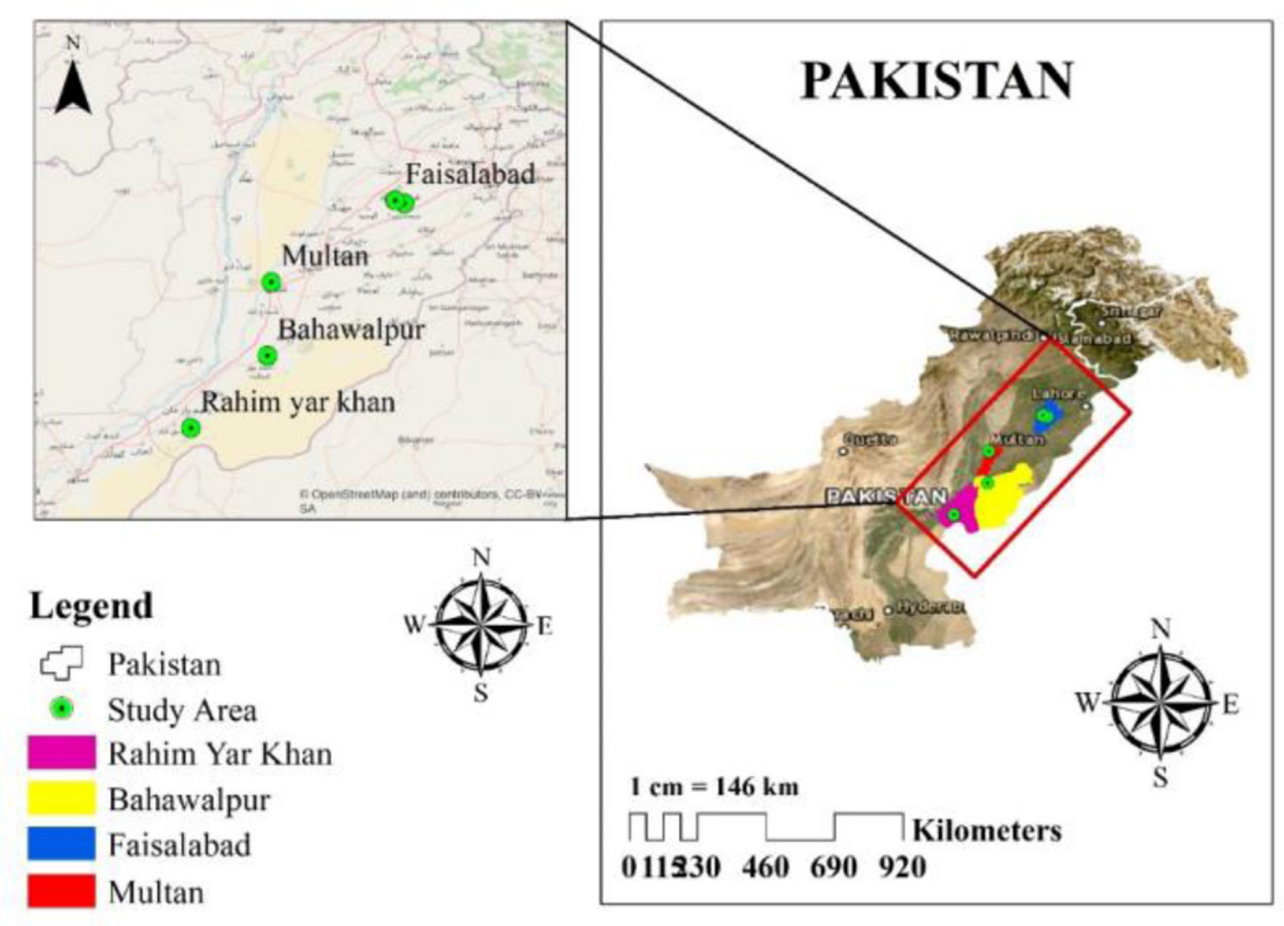
Location map showing soil sampling sites for the isolation of glyphosate-tolerant bacteria.

Four cities in Punjab, Pakistan, were chosen to collect rhizosphere samples (December 2019–March 2020), i.e., Rahim Yar Khan, Multan, Bahawalpur, and Faisalabad, with a 5- to 10-year history of GP usage as shown in Figure 1. The randomly collected soils were stored in zip lock bags at 4°C until further use. The physical and chemical characteristics of rhizosphere soil utilized for isolation and screening of GP-tolerant rhizobacterial strains are shown in Table 1.

**Table 1:** Characteristics of the physical and chemical conditions of the rhizosphere samples from which glyphosate-degrading bacteria were isolated.

**Location**	**Source of isolation**	**EC (dS m^−1^)**	**pH**	**CEC (cmol kg^−1^ soil)**	**Organic matter (%)**	**Moisture content (%)**
Rahim Yar Khan	Wheat	1.67	8.3	9.17	0.55	39
Rahim Yar Khan	Maize	1.82	7.6	10.04	0.52	31
Rahim Yar Khan	Cotton	2.11	7.5	10.87	0.61	50
Faisalabad	Maize	2.43	7.8	8.52	0.47	42
Faisalabad	Wheat	1.91	8.3	11.47	0.76	47
Faisalabad	Wheat	2.11	7.6	10.62	0.69	45
Faisalabad	Maize	1.73	8.4	9.53	0.83	48
Bahawalpur	Maize	5.31	8.6	10.15	0.41	45
Bahawalpur	Cotton	4.78	8.1	12.54	0.39	40
Multan	Maize	3.53	7.7	10.84	0.66	48
Multan	Wheat	3.65	8.7	9.87	0.57	42

Eleven bacterial strains (WAG1–WAG11) were isolated from different soils amended with glyphosate 200 mg kg^−1^. Soils came from four different locations where wheat, maize, or cotton was grown.

### 2.2 Isolation and purification of bacterial strains by enrichment culture technique

Glyphosate-resistant bacterial strains were isolated via an enrichment culture technique using GP as the C source. Collected samples (5 g soil) were transferred in mineral salt medium (MSM) for this experiment (Mbagwu et al., 2019) consisting of KH_2_SO_4_, 3 g; NaCl, 0.5 g; NH_4_Cl, 1 g; Na_2_SO_4_, 5.8 g; and MgSO_4_.7H_2_O, 0.25 g, and pH 7.0 was maintained in distilled water enriched with Focht trace element solution comprising GP (100 mg L^−1^) for 14 days at 28°C and shaken at 130 rpm in an incubator. In total, 1 mL of inoculum was transferred to MSM (freshly prepared) after 14 days with GP (150 mg L^−1^) and again put into a shaker for 14 days with the conditions mentioned above. The procedure mentioned above was recurring, although the GP concentration was boosted to 200 mg L^−1^. A total of 300 μL of inoculant was poured on an MSM agar plate after 14 days using the serial dilution technique and incubated at 28 ± 2°C for 48 h in the aerobic environment to obtain GP-resistant bacterial strains.

### 2.3 Exploring the characteristic of glyphosate-tolerant bacteria for plant growth

**Figure 2:**
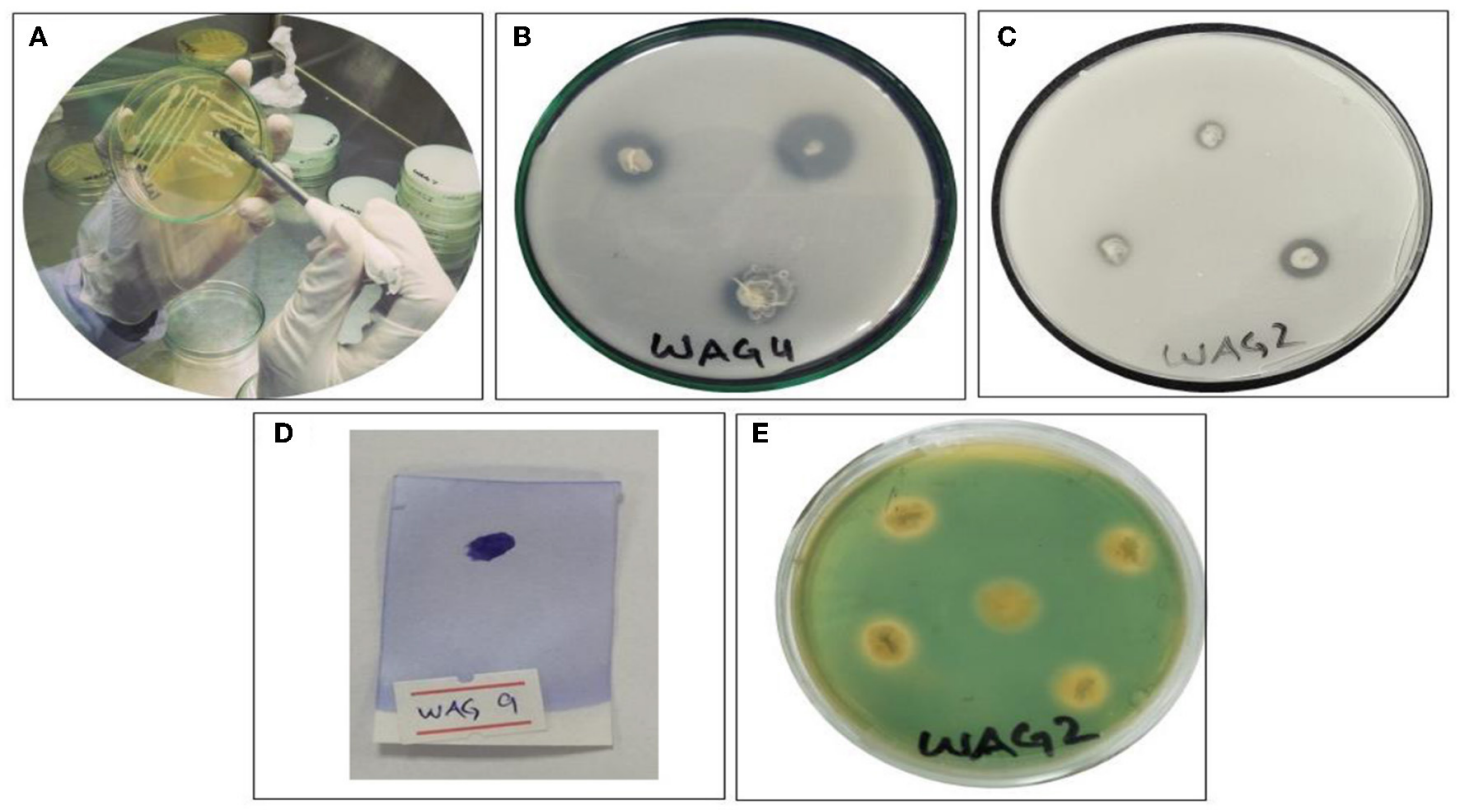
Screening of bacterial isolates for the plant growth promotion assay. **(A)** Screening and purification of glyphosate-resistant bacterial strains, **(B)** phosphorous solubilization, **(C)** chitinase solubilization, **(D)** oxidase positive, and **(E)** siderophore production.

The qualitative plant growth promotion characteristics of isolated bacterial strains were determined using the following protocols. General-purpose media were used to determine the indole-3-acetic acid (IAA) (Zhao et al., 2021). The level of oxidase activity was determined by the protocol followed by Lysenko (1961). Siderophores production was determined using the method followed by Manwar et al. (2004). The National Botanical Research Institute's phosphate (NBRIP) agar media were used to observe the P solubilization followed by Collavino et al. (2010). Chitinase activity was observed by the strategy described by Ashraf et al. (2004) as shown in Figure 2. Catalase production was determined by the method followed by Guettler et al. (1999). Exopolysaccharide production was determined by the method followed by Ashraf et al. (2004). Stress-coping 1-aminocyclopropane-1-carboxylic acid (ACC) deaminase was analyzed by the technique followed by Mehta et al. (2015). The colonization of microbes test in the root was done via the method followed by Hanif et al. (2014). To identify the isolated bacterial strains, fresh cultures (24–48 h) were sent to Macrogen in Korea. A specific primer was utilized to amplify the 16S ribosomal rRNA: forward and reverse primers consisted of the following sequence: ‘27F 5' (AGA GTT TGA TCM TGG CTC'AG) 3' and 1‘92R 5' (TAC GGY TAC CTT GTT ACG AC' T) 3'. The obtained sequence was submitted to NCBI to create the accession number. The evolutionary history was inferred by the neighbor-joining method. The sequences were aligned using Mega X computer-based software. A phylogenetic tree in the circular form of all bacterial strains was constructed using the Interactive Tree of Life online tool to display and manage phylogenetics (Letunic and Bork, 2007) to provide light on the evolutionarily based connection between strains and their close proximate species.

### 2.4 Chemicals and reagents

All the chemicals were of analytical grade. Glyphosate (99.7%), FMOC-Cl (97%), acetonitrile, and diethyl ether were purchased from Sigma Aldrich^®^ (Seelze, Germany).

### 2.5 Analytical process of glyphosate detection

Glyphosate spiked soil (10 g) was taken from an earthen pot and added into a 50-ml centrifuge tube following the addition of 20 mL 0.01 M KH_2_PO_4_ and put into a rotary shaker for 2 h. This process is followed by 10 min of centrifugation for 10,000 RPM at 4°C. Supernatants were filtered through a 0.22-μm syringe filter (Garba et al., 2018). The derivatization process was done by adding a 1-mL syringe filter solution in a centrifuge tube (25 mL). Then, 1 mL of 0.02 M FMOC-Cl and 2 mL of 0.05 M borate buffer were mixed with 1 mL of syringe filter solution. The solution was agitated for an hour on an end-to-end shaker at 180 RPM. To get rid of any unreacted FMOC-Cl, 2 mL of diethyl ether was poured into the test tube and then vortexed for 2 min. After removing the organic layer, the obtained aliquots were poured into vials (GC) for further estimation.

The content of GP was analyzed by Sykam High-Pressure Liquid Chromatography (HPLC) system (Germany) integrated with UV/Vis Diode Array Detector (Model S 3345), pump (Model S1125G), and column C18 and column oven (Model S 4120). HPLC quality acetonitrile and KH_2_PO_4_ (0.05 M) (30:70 v/v) mobile phase were utilized in an isocratic approach. The pump ran at 0.7 ml min^−1^ for 15 min. The insertion volume was 20 μL, and the temperature of the column was 40°C. Two wavelengths, 210 and 315 nm, were used to determine GP residue through the HPLC. GP had a retention time of 3 min. Clarity chromatography software was used to collect and analyze the data. Values were obtained by interpolating using a calibration curve made from GP standards of known concentrations (Garba et al., 2018). The Eq. (1) measured GP biodegradation after 7, 14, and 28 days of application (1).


(1) $$\begin{array}{l} Glyphosate\;degraded\;mg\;kg^{-1}=\;T_{0}-T_{1} \end{array}$$

where T_0_ represents GP concentration at 0 h, and T_1_ represents glyphosate concentration in the sample.

### 2.6 Glyphosate degradation and plant growth under ambient conditions

The study was performed at the University of Agriculture Faisalabad (UAF), Pakistan. Soil (pesticide-free) samples were collected at 0 to 15 cm depth at three randomly selected sites and mixed thoroughly. The wet soil was sieved (4 mm) to remove plant material and coarse rock, thoroughly mixed to maintain homogeneity, and stored at 4°C. A sub-sample of almost half a kilogram was taken, air-dried, passed through a 2-mm sieve, and treated to determine physicochemical properties as presented in Table 2. Pre-analysis of soil was done to check the GP residue according to the standard protocol. Soil pH was observed in D.I. water with a glass electrode (soil:1 and H_2_O:2.5 w/v). The hydrometer technique analyzed the soil texture, while the Kjeldahl method observed total nitrogen (N) in the soil (Selassie et al., 2015). The organic matter was assessed using a Mebius technique (Yeomans and Bremner, 1988). Soil P and K were quantified by the methods explained by Abbasi and Khizar (2012).

**Table 2:** Physico-chemical properties of the soil used in pot experiments.

**Parameters**	**Soil**
Sand (%)	52.0
Silt (%)	32.9
Clay (%)	15.1
CEC (cmol kg^−1^)	6.2
pH	7.8
Organic C (%)	0.04
Organic matter (%)	0.08
C:N ratio	10.1
Total mineral N (%)	0.8
K (%)	8.8
P (%)	0.35

### 2.7 Pot experiment

A pot experiment was conducted in the wirehouse of the Institute of Soil and Environmental Sciences, UAF, Pakistan, to evaluate the indigenous GP-degrading bacteria at different concentrations during maize growth. Various morphological and physiological characteristics of maize and soil enzymatic activities were also recorded, along with biodegradation efficiency. Thoroughly cleansed earthen (clay) pots of height (38 cm) and width (18 cm) were utilized in this experiment. Each pot was filled with 12 kg of soil, and the polyethylene bag was lined inside the earthen pot to avoid leaching. Hybrid maize seeds (Pioneer-1543) were procured from Corteva Agriscience and treated with 95% ethanol and HgCl_2_ (0.2%) for at least 3 min (Shaharoona et al., 2007). For the maize (*Zea mays*) seed planting with eight plants per pot, 5 mL of a bacterial inoculum with a cell density of 10^−7^ CFU mL^−1^ was prepared. Glyphosate 100 and 200 mg kg^−1^ were mixed in water and poured into the soil to reach the GP concentration at 100 and 200 mg kg^−1^. The soil saturation was done with drinking water and retained water occupied (58%) pore space. In every earthen pot, eight seeds were sown at 3 cm depth during the summer season of July 2020. Earthen pots were kept under cover to lower evapotranspiration in the germination process. After germination, the plants were thinned to one plant in each pot. All pots were uniformly watered when required. The pot trial was performed in two sets with a subsequent layout: (1) control (without inoculation) spiked with GP (100 mg L^−1^ and 200 mg L^−1^), (2) GP-resistant bacterial strains (inoculation) applied separately in each earthen pot spiked with GP (100 mg L^−1^ and 200 mg L^−1^). Three independent sets of each treatment were set up in a completely randomized design (CRD) (Gomez and Gomez, 1984). The experiment lasted for 120 days in the wirehouse under natural conditions. The average temperature was 41°C and 10 h of daylight. Soil samples were taken at different intervals of days to check the GP biodegradation according to standard procedure (Mohy-Ud-Din et al., 2023).

### 2.8 Seed germination index

The seed germination index was calculated using the following formula (Marcu et al., 2013):


$$\begin{array}{l} GI\;\left(\%\right)=\frac{Tg}{Tt}\times 100 \end{array}$$

where Tg is the germinated seeds, and Tt is the total number of seeds that were sown.

### 2.9 Determination of morphological and physiological characteristics

Morphological characteristics such as shoot and root fresh weight and length were assessed after harvesting (120 days). Physiological parameters, i.e., relative water contents and total protein contents, were recorded 70 days after the germination of maize.

### 2.10 Determination of relative water content

Saghafi et al. (2018) used the following formula for analyzing the relative water content, as shown in Eq. (2).


(2) $$\begin{array}{l} Relative\;water\;content\;\left(RWC\right)=\left(FW-DW\right)÷\left(FTW-DW\right) \end{array}$$

where FW represents fresh weight, DW represents dry weight, and FTW represents fully turgid weight. The fresh leaf attained 100% moisture after 48 h at 4°C and is designated as a fully turgid weight.

### 2.11 Determination of total protein content

The Bradford assay method was used to determine protein concentration (Bradford, 1976; Vardharajula et al., 2011) on Thermo Scientific Evolution 300 UV-Vis Spectrophotometers at Soil Microbiology and Biochemistry lab, University of Agriculture Faisalabad, Pakistan. The 200 μL of leaf extract was mixed with 1800 μL of D.I. water and 2 mL of Bradford reagent. After 10–20 min of incubation at 28°C, the samples and blank samples were read for absorbance at 595 nm. To quantify the amount of protein, a standard curve technique was used. The concentrations used to create the standard curve ranged from 0 to 300 ppm. To prepare the Bradford reagent, 50 mg of Coomassie Blue G-250 was dissolved in methanol (50 mL). After that, 100 mL of phosphoric acid at 85% concentration was added to the solution, and the whole thing was combined with 500 mL of deionized water and filtered to get rid of any leftover residues. This solution was further diluted with 350 mL of D.I. water and kept at 4°C for later use.

### 2.12 Determination of proline and malondialdehyde in maize leaves

Extraction of plant enzyme was done with pestle and mortar (pre-chilled) to homogenize leaf (maize) samples in a pH 7.0 phosphate buffer following the procedure by Shabaan et al. (2022). The mixture was processed through centrifugation (cooled condition) at 9000 rpm, and malondialdehyde (MDA) and proline contents were evaluated by following the protocols. Proline content analysis was done according to the process by Bates et al. (1973). A plant sample 0.5 g was homogenized in sulfosalicylic acid and then filtered the mixture. The filtrate was mixed with 2 mL of acid ninhydrin and 2 mL of glacial acetic acid; a frost bath was later applied to stop the reaction. Extraction of the sample from the reaction mixture was done with the help of toluene. The extracted mixture absorbance was measured on a spectrophotometer at 520 nm. A standard curve was constructed to measure the proline contents (Bates et al., 1973).

Malondialdehyde concentration in maize leaves was analyzed using the method followed by Shabaan et al. (2022). A total of 10% of trichloroacetic acid (10%) was used to homogenize the leaf samples, followed by centrifugation at 9,000 rpm for 20 min. A reaction mixture comprising 2 ml of sample, thiobarbituric acid, and trichloroacetic acid (0.6 and 10%) was heated at 100°C for 15 min. After that, the reaction mixture was cooled down in an ice bath to stop the reaction. Again, the reaction mixture was centrifuged at 9,000 rpm for 20 min, and supernatant absorbance was done by spectrophotometer at 450 and 532 nm. Malondialdehyde concentrations were expressed as nmol min^−1^ mg^−1^ protein.

### 2.13 Determination of enzymatic activities in soil

To assess acid, alkaline phosphatase, and dehydrogenase activities in rhizosphere soil, samples were collected, stored at −4°C, and analyzed using established methods. Acid and alkaline phosphatase activities were determined following the procedure described by Eivazi and Tabatabai (1977), involving colorimetric assessment of p-nitrophenol production in soil incubated with a p-nitrophenyl phosphate (PNPP) solution and toluene. Standard buffers were prepared for both acidic (pH 6.5) and alkaline (pH 11) phosphatase, and the activity was measured at 410 nm using a spectrophotometer. Dehydrogenase activity in rhizosphere soil was quantified by colorimetric measurement of 1, 3, 5-triphenyl formazan (TPF), employing a method developed by Lenhard (1956). This involved the reduction of 2, 3, 5-triphenyl tetrazolium chloride (TTC) by soil-inoculated GP-tolerant bacteria. The absorbance of the resulting reddish color was assessed at 485 nm using a spectrophotometer, with methanol as a blank sample. A standard curve derived from known standards was utilized to calculate dehydrogenase production, expressed as mg TPF produced per hour per gram of dry weight of soil.

### 2.14 Statistical analysis

The experiment consisted of 24 treatments with triplicates. Microsoft Excel Worksheet (version 2205, US) calculated treatment means, standard deviation, and standard errors. The obtained GP biodegraded concentration, plant growth promotion, plant antioxidants, and soil enzyme activity difference among treatment means were calculated by Tukey's honestly significant difference (HSD) test at *p* > 0.05 in OriginPro 2022b. Furthermore, Pearson's correlation (PC) among biodegradation of GP, plant growth promotion, plant antioxidants, and soil enzymatic activities was accomplished in RStudio. The built-in function created a heat map matrix from the publicly accessible package “corrplot” as per R-project commands (Team, 2019).

## 3 Results

### 3.1 Screening of glyphosate-degrading bacteria

Through 10 separate growth-promoting (GP) enrichments across various soil samples, we successfully isolated 11 distinct bacterial strains. It is noteworthy that, in most instances, only a limited number of colonies exhibited growth on individual culture plates (Table 1). To explore their potential to enhance plant development, we subjected these strains to comprehensive characterization, evaluating their capabilities in exopolysaccharide production, 1-aminocyclopropane-1-carboxylic acid (ACC) utilization, catalase and oxidase activities, indole acetic acid production, siderophore secretion, phosphorus solubilization, root colonization, and chitinase production (Mohy-Ud-Din et al., 2023). Initially, **t**hese 11 rhizobacterial strains were designated as WAG1-WAG11.

### 3.2 Comparison of glyphosate degradation efficiency of selected strains

**Figure 3:**
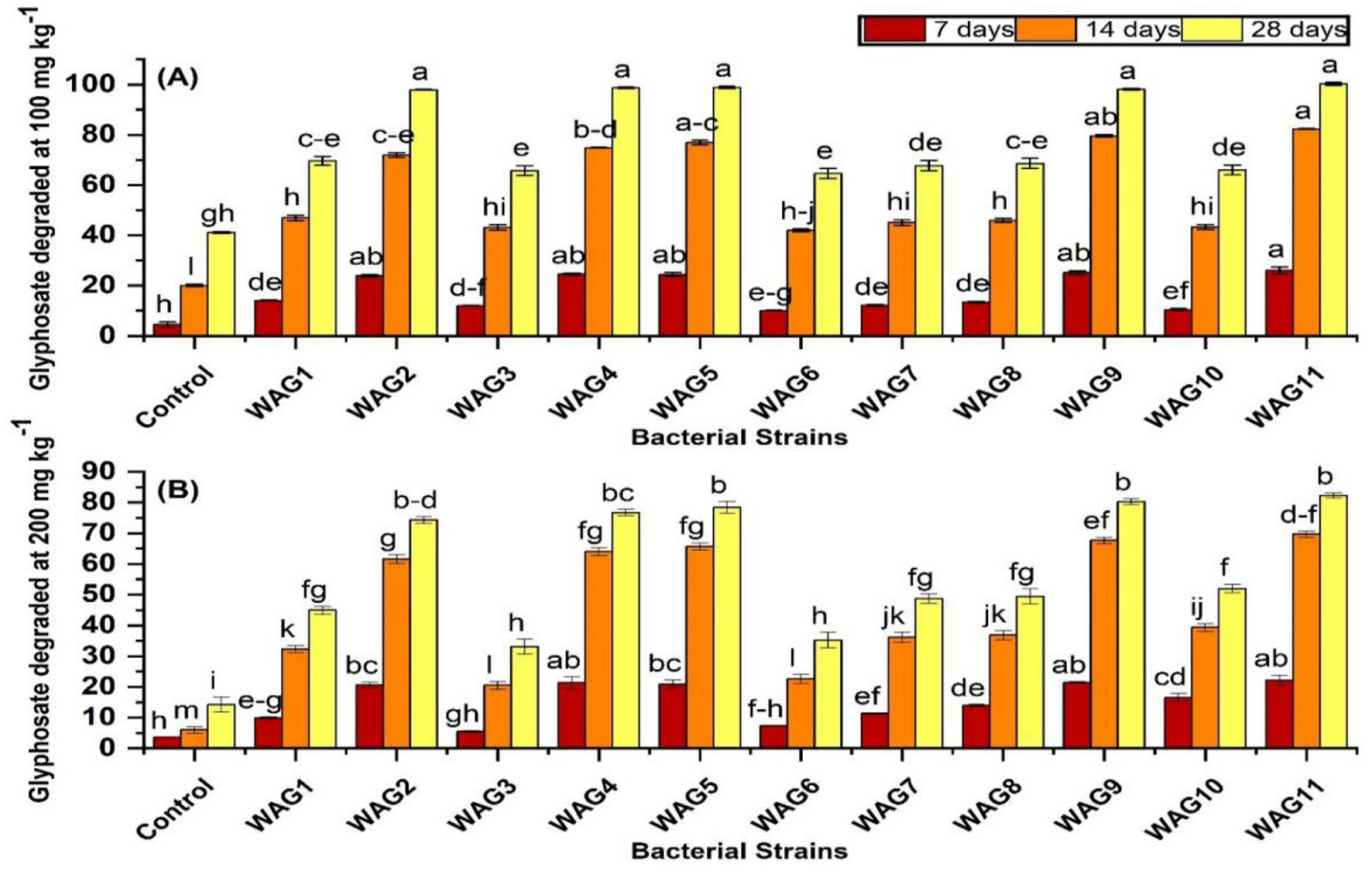
Degradation of glyphosate by isolated bacteria in soil containing 100 mg kg^−1^ glyphosate **(A)** and 200 mg kg^−1^ glyphosate **(B)** in 7, 14, and 28 days, respectively. The presented data reflect the average of triplicate measurements, with the error bars representing the standard deviations. There is no significant difference between bars that share the same letter (s) at a *p* < 0.05.

Based on the results, significantly higher GP biodegradation was observed in biotically inoculated soil compared to the abiotically uninoculated control soil. To assess the efficiency of GP biodegradation, we evaluated 11 bacterial strains under two GP concentrations (100 and 200 mg kg^−1^) (Figures 3A, B). Notably, under both GP concentrations, strains WAG11, WAG9, WAG5, WAG4, and WAG2 exhibited the highest GP degradation levels across three time points (7, 14, and 28 days) (Figure 2A). Strain WAG11 achieved complete GP degradation at 100 mg kg^−1^ and 36.7% degradation at 200 mg kg^−1^ after 28 days. WAG5 exhibited 98% and 34% degradation at 100 and 200 mg kg^−1^, respectively after 28 days. Similarly, WAG4 exhibited 97% and 33.7% GP degradation at 100 and 200 mg kg^−1^, respectively. WAG9 and WAG2 both achieved 96% GP degradation at 100 mg kg^−1^, with WAG9 degrading 35% and WAG2 degrading 32% at 200 mg kg^−1^ after 28 days. In contrast, WAG3 and WAG6 exhibited minimal GP degradation, with 39.9 and 11% at 100 mg kg^−1^ and 41 and 10.2% at 200 mg kg^−1^, respectively after 28 days. In the uninoculated control (abiotically uninoculated) condition, GP degradation reached 41.12 (41%) and 14.16 (7%) mg kg^−1^ at 100 and 200 mg kg^−1^ after 28 days of application. The inoculation of GP-resistant pure bacterial strains significantly enhanced GP decomposition in soil.

### 3.3 Effect of the bacterial supplement on seed germination and shoot and root length of maize

Compared to the uninoculated control, the germination rate index and root emergence were dramatically improved by inoculation with the appropriate bacterial strains in the presence of 100 and 200 mg kg^−1^ of GP. In comparison with their respective controls, the germination rates of maize seed in the WAG11, WAG9, WAG5, WAG4, and WAG2 inoculated treatments on average improved by 71% at 100 mg kg^−1^, but, at 200 mg kg^−1^ of GP, inoculation of WAG2, WAG4, WAG5, WAG9, and WAG11 strains exhibited 80% improved germination than the respective uninoculated control, as presented in Table 3.

**Table 3:** Inoculating effect on seed germination rate in two different concentrations of glyphosate.

	**Seed germination (%)** ^**a**^	
**Bacterial strains**	**100 mg kg** ^−1^	**200 mg kg** ^−1^
Control	4 ± 0 c–e	3 ± 0 e
WAG1	6 ± 0 bc	4 ± 0 de
WAG2	8 ± 0 a	6 ± 1 bc
WAG3	6 ± 1 bc	5 ± 0 cd
WAG4	8 ± 0 a	6 ± 0 bc
WAG5	7 ± 0 ab	6 ± 0 bc
WAG6	6 ± 0 bc	5 ± 0 cd
WAG7	6 ± 0 bc	4 ± 1 de
WAG8	4 ± 0 de	4 ± 0 de
WAG9	8 ± 0 a	6 ± 0 bc
WAG10	5 ± 0 cd	4 ± 1 de
WAG11	8 ± 0 a	6 ± 0 bc
HSD value (*p* ≤ 0.05)	1.4673	

a The results signify the mean value of triplicates, and ± represents standard deviations. Same letters shared by means do not differ significantly at a p-value of < 0.05.

### 3.4 Effect of bacterial inoculation on maize morphology

A significant impact of PGPR inoculation was measured in different parameters, such as increases in the root and shoot fresh weight. It is usually accepted that the presence of microbial colonies in the rhizosphere is an essential stage for observing the positive impact of microbes on plants (Riaz et al., 2021). The association of isolated bacterial strains was examined with maize plants under GP-spiked soil with two concentrations (100 and 200 mg kg^−1^). The average increase of shoot fresh weight was observed at 73.88% by strains (WAG2, WAG4, WAG5, WAG9, and WAG11) compared to uninoculated control at 100 mg kg^−1^ of GP. At 200 mg kg^−1^ of GP, the average increase of shoot fresh weight by respective bacteria was 81.85% more than the respective control. The results revealed that GP at both concentrations (100 and 200 mg kg^−1^) significantly reduced the shoot and root length in control (uninoculated condition).

**Figure 4:**
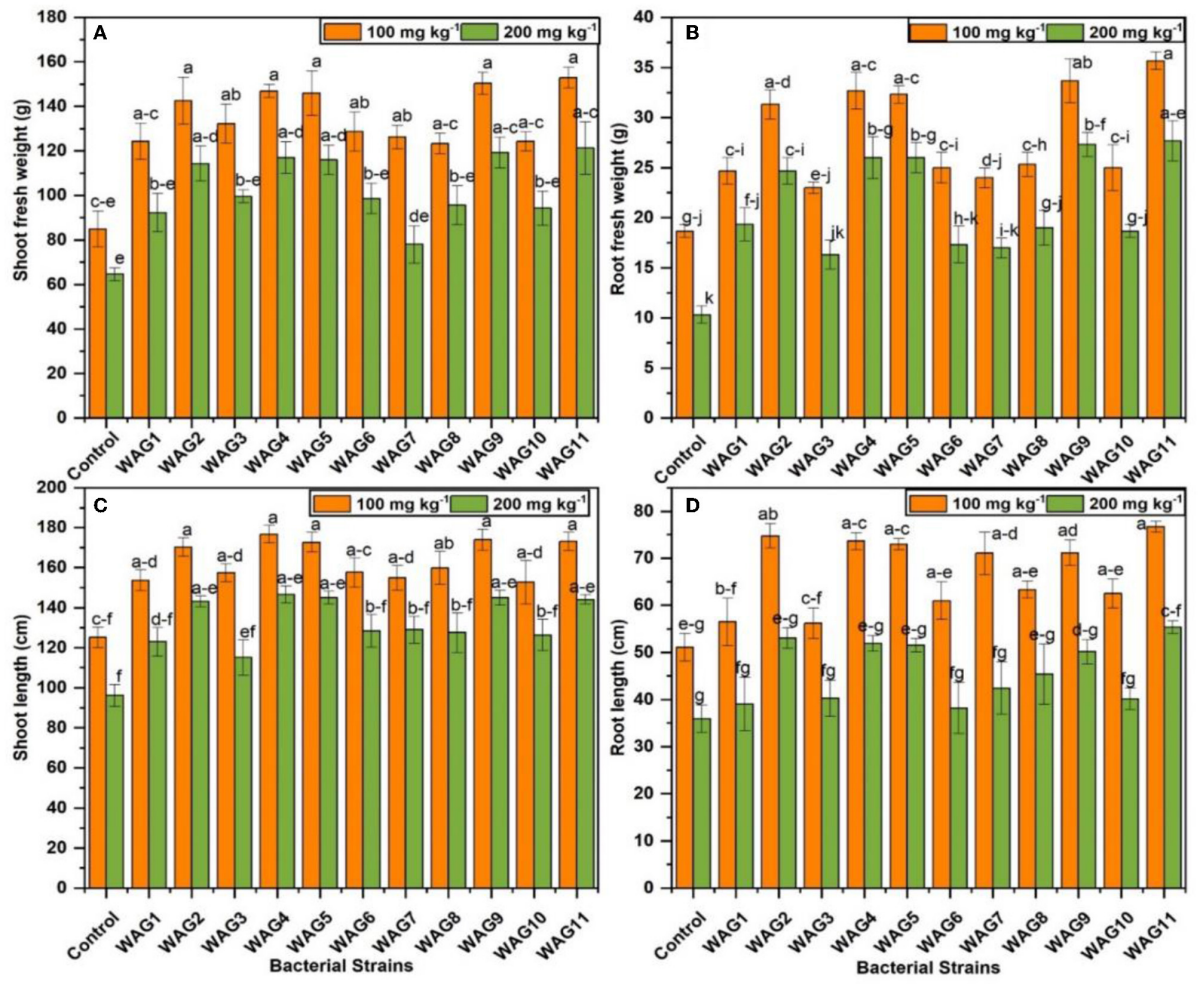
Maize growth parameters under eleven isolated bacteria at 100 and 200 mg kg^−1^ glyphosate in soil; the control contained no bacteria. **(A)** Shoot fresh weight (g), **(B)** root fresh weight (g), **(C)** shoot length (cm), and **(D)** root length (cm). The data shown are the mean of three replicates, with the error bars displaying the standard deviations. The same letters shared by bars do not differ significantly at a *p* < 0.05.

In comparison, inoculation of GP-tolerant bacterial strains improved the plant growth parameters at both concentrations than the control (uninoculated). The highest shoot length observed by the WAG9 inoculated treatment was 39 and 50%, respectively, more than the respective uninoculated control at 100 and 200 mg kg^−1^ of GP. In the case of root, the highest root length was observed at 50 and 54% in treatments inoculated by the WAG11 at both levels of GP, as shown in Figure 4. The root is a significant part of a plant that helps to provide nutrients to the upper part of the plant body. The maximum root fresh weight was improved by 91% by inoculation of WAG11, while the WAG9 strain was enhanced by 80% compared to the control at 100 mg kg^−1^ of GP. The lowest increase in root fresh weight was seen by the WAG3 strain, which was 23% (Figure 4), though at 200 mg kg^−1^ of GP, both bacterial strains WAG9 and WAG11 improved 1.64- to 1.67-fold more root fresh weight than their respective control (uninoculated conditions). The minimum increase (58%) of root fresh weight was observed by WAG3. As discussed above, the results indicated that inoculation of GP-tolerant bacterial strains significantly improved growth promotion characteristics under different concentrations of GP.

### 3.5 Effect of isolated bacterial inoculation on maize physiology

**Figure 5:**
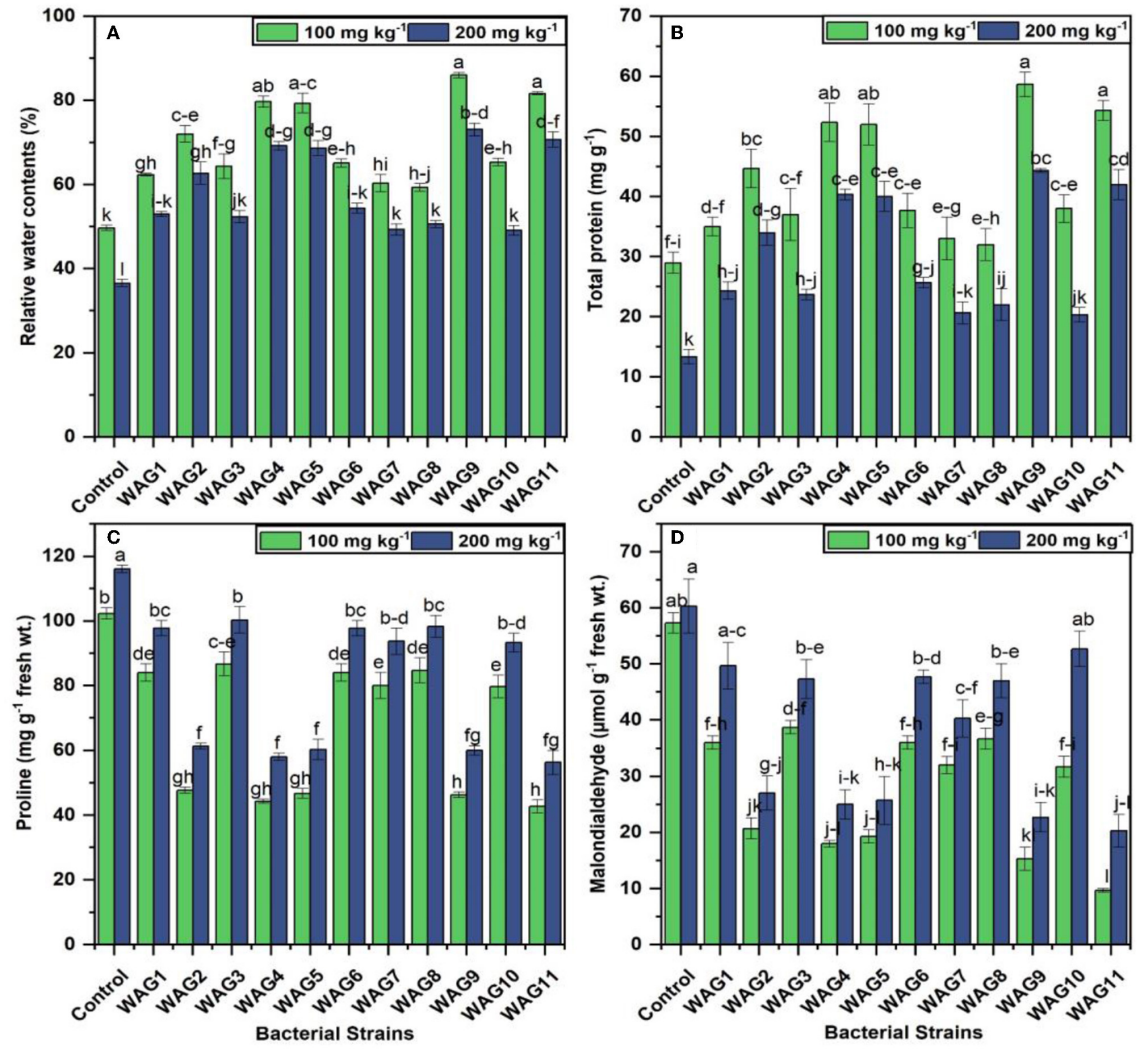
Maize stress copping parameters under eleven isolated bacteria at 100 and 200 mg kg^−1^ glyphosate in soil; the control contained no bacteria. **(A)** Relative water contents (%), **(B)** total protein (mg g^−1^), **(C)** proline (mg g^−1^ fresh wt.), and **(D)** malonaldehyde (μmol g^−1^ fresh wt.). The data shown are the mean of three replicates, with the error bars displaying the standard deviations. The same letters shared by bars do not differ significantly at a *p* < 0.05.

The application of rhizobacterial strains significantly improved the relative water content compared to the control at 100 and 200 mg kg^−1^. Inoculated treatment, i.e., WAG9, significantly improved 73 and 99% of relative water contents at 100 and 200 mg kg^−1^ of GP, respectively compared to their respective control (uninoculated condition) presented in Figure 5. While the least improvement was observed by WAG8, 19% at 100 mg kg^−1^, and WAG10 exhibited 33% at 200 mg kg^−1^ of GP than the uninoculated control, the GP-tolerant bacterial strains inoculation showed considerably enhanced total protein contents in maize leaves at both levels of GP (100 and 200 mg kg^−1^). Isolated bacterial strain WAG9 revealed 102% and WAG11 observed 87% improved total protein contents in maize leaves compared to the control (uninoculated condition). In contrast, the minimum improvement was observed by WAG8, WAG7, and WAG1, which were 10, 13, and 20%, respectively, at 100 mg kg^−1^ after 70 days of sowing. At 200 mg kg^−1^ concentration of GP, the highest total protein contents were improved in the following order WAG9, WAG11, WAG4, and WAG5, that was 232, 215, 202, and 200%, respectively, as compared to the control (uninoculated condition) after 70 days of sowing in Figure 5. However, minimum improvement was observed by WAG10, which was 52% as compared to the control (uninoculated condition).

### 3.6 Determination of proline and malonaldehyde in plants

The bacterial strain inoculation significantly reduced the proline due to GP stress at 100 and 200 mg kg^−1^ on maize plants than to control (uninoculated condition) after 70 days of sowing. The results revealed that proline and MDA production under two different concentrations of GP significantly improved the proline and MDA production in the control (uninoculated) treatments, i.e., the observed proline contents (102.3 and 116 μmole g^−1^ fresh weight) and MDA (57 and 60 μmole g^−1^ fresh weight), respectively, at 100 and 200 mg kg^−1^ of GP presented in Figure 5. Similarly, among all the inoculated treatments, the highest reduction of proline contents was observed by inoculation of WAG11 (42 and 56 μmole g^−1^ fresh weight) and MDA (9 and 20 μmole g^−1^ fresh weight), respectively, at 100 and 200 mg kg^−1^. However, the minimum reduction of proline contents in inoculated treatment was observed by WAG3 (86 and 100 μmole g^−1^ fresh weight) and MDA (31 and 52 μmole g^−1^ fresh weight), respectively, at both concentrations in Figure 5.

### 3.7 Determination of enzymatic activities in soil

In soil enzymatic activities (acid, alkaline phosphatase, and dehydrogenase), the results revealed that GP toxicity significantly decreased the soil enzymatic activity in control (uninoculated) at both concentrations of GP (100 and 200 mg kg^−1^). Maximum reduction of soil enzyme was observed in the control that was 88 and 93 acid phosphatase μmol PnP g^−1^ soil h^−1^, 17 and 20 alkaline phosphatases μmol PnP g^−1^ soil h^−1^, and 5 and 4 dehydrogenase μmole TPF mg^−1^ soil h^−1^, respectively, at 100 and 200 mg kg^−1^. Maximum improvement was observed by inoculation of WAG11 (61%) and WAG9 (59%) at 100 mg kg^−1^ of GP than the respective control in Table 4, while at 200 mg kg^−1^ of GP, application of WAG11 (69%) and WAG9 (67%) showed improved acid phosphatase activity in soil as compared to the respective control (uninoculated). Similarly, inoculation of WAG2 (167%) and WAG5 (169%) showed more alkaline phosphatase than the control at 100 mg kg^−1^. Bacterial strains WAG2 and WAG5 showed 153% and 156% more alkaline phosphatase activity than the respective control at 200 mg kg^−1^ of GP in Table 4. Interestingly, alkaline phosphatase improvement was more at 200 mg kg^−1^ compared to 100 mg kg^−1^ of GP. In dehydrogenase activity in the soil, excessive doses of GP (100 and 200 mg kg^−1^) significantly reduce the dehydrogenase activity in control (uninoculated) soil. Through inoculation of GP-resistant WAG5 and WAG9 strains, dehydrogenase activity was considerably increased by 188% and 182% as compared to the respective controls at 100 mg kg^−1^ of GP. At 200 mg kg^−1^ of GP, WAG5 and WAG9 significantly boost 2.5-fold and 2.3-fold, respectively, dehydrogenase activity in soil presented in Table 4. Minimum dehydrogenase activity was observed by treatment inoculated with WAG3 (17 and 23%), respectively, at both levels of GP.

**Table 4:** Effect of isolated rhizobacteria on soil enzymatic activities under glyphosate spiked soil.

	**Acid Phosphatase**^**a**^ **(**μ**mol PnP g**^**−1**^ **soil h**^**−1**^**)**		**Alkaline Phosphatase**^**a**^ **(**μ**mol PnP g**^**−1**^ **soil h**^**−1**^**)**		**Dehydrogenase**^**a**^ **(**μ**mole TPF mg**^**−1**^ **soil h**^**−1**^**)**	
**Bacterial strains**	**100 mg kg** ^−1^	**200 mg kg** ^−1^	**100 mg kg** ^−1^	**200 mg kg** ^−1^	**100 mg kg** ^−1^	**200 mg kg** ^−1^
Control	88.33 ± 1.76 k	93.67 ± 4.31 k	17.33 ± 1.2 h	20.67 ± 0.88 gh	5.67 ± 0.66 de	4.33 ± 0.88 e
WAG1	115 ± 2.3 ij	127.33 ± 1.85 f-h	29 ± 1.15 d-g	33.67 ± 0.88 d	7.33 ± 0.88 c-e	5.67 ± 0.33 de
WAG2	137.33 ± 2.32 c-f	153.67 ± 3.83 ab	46.33 ± 0.66 c	52.33 ± 0.33 a-c	15.33 ± 1.76 a	13.33 ± 1.2 ab
WAG3	108 ± 0.57 j	121.33 ± 1.45 g-i	26.33 ± 2.39 d-g	31 ± 2.63 d-e	6.67 ± 1.2 c-e	5.33 ± 1.45 e
WAG4	139.67 ± 0.88 c-e	156 ± 1.52 a	47.33 ± 1.2 bc	53.33 ± 2.32 a-c	15.33 ± 0.66 a	13.67 ± 1.33 ab
WAG5	137.67 ± 1.76 c-f	154 ± 0 ab	46.67 ± 0.88 c	53 ± 1 a-c	16.33 ± 1.45 a	15.67 ± 0.33 ab
WAG6	119.33 ± 2.02 h-j	132.33 ± 2.72 c-g	24 ± 1 e-h	28.67 ± 0.88 d-g	7.33 ± 0.88 c-e	6.67 ± 1.2 c-e
WAG7	118.33 ± 1.76 h-j	129 ± 1.72 d-h	23.67 ± 2.39 e-h	28.33 ± 1.2 d-g	10.33 ± 1.2 bc	10.33 ± 1.45 bc
WAG8	117.67 ± 2.18 h-j	129 ± 1.15 d-h	25 ± 1.15 d-h	29.67 ± 1.85 d-f	9.33 ± 0.33 cd	7.33 ± 0.88 c-e
WAG9	140.67 ± 1.85 cd	157 ± 2.07 a	48.67 ± 2.18 a-c	56 ± 2.63 ab	16 ± 1.15 a	15.33 ± 1.76 a
WAG10	118.33 ± 2.39 h-j	128.33 ± 4.65 e-h	21.33 ± 2.39 f-h	26 ± 1.52 d-h	8 ± 0.57 c-e	6.67 ± 0.33 c-e
WAG11	142.33 ± 1.76 bc	158.67 ± 0.66 a	51 ± 1.15 a-c	57.33 ± 0.88 a	15 ± 1 a	14 ± 0.57 a
HSD value (*p* ≤ 0.05)	12.030		8.9696		3.9221	

a The results correspond to the mean value ± standard deviation of triplicates, and same letters shared by means do not differ significantly at a p < 0.05.

### 3.8 Correlation analysis

**Figure 6:**
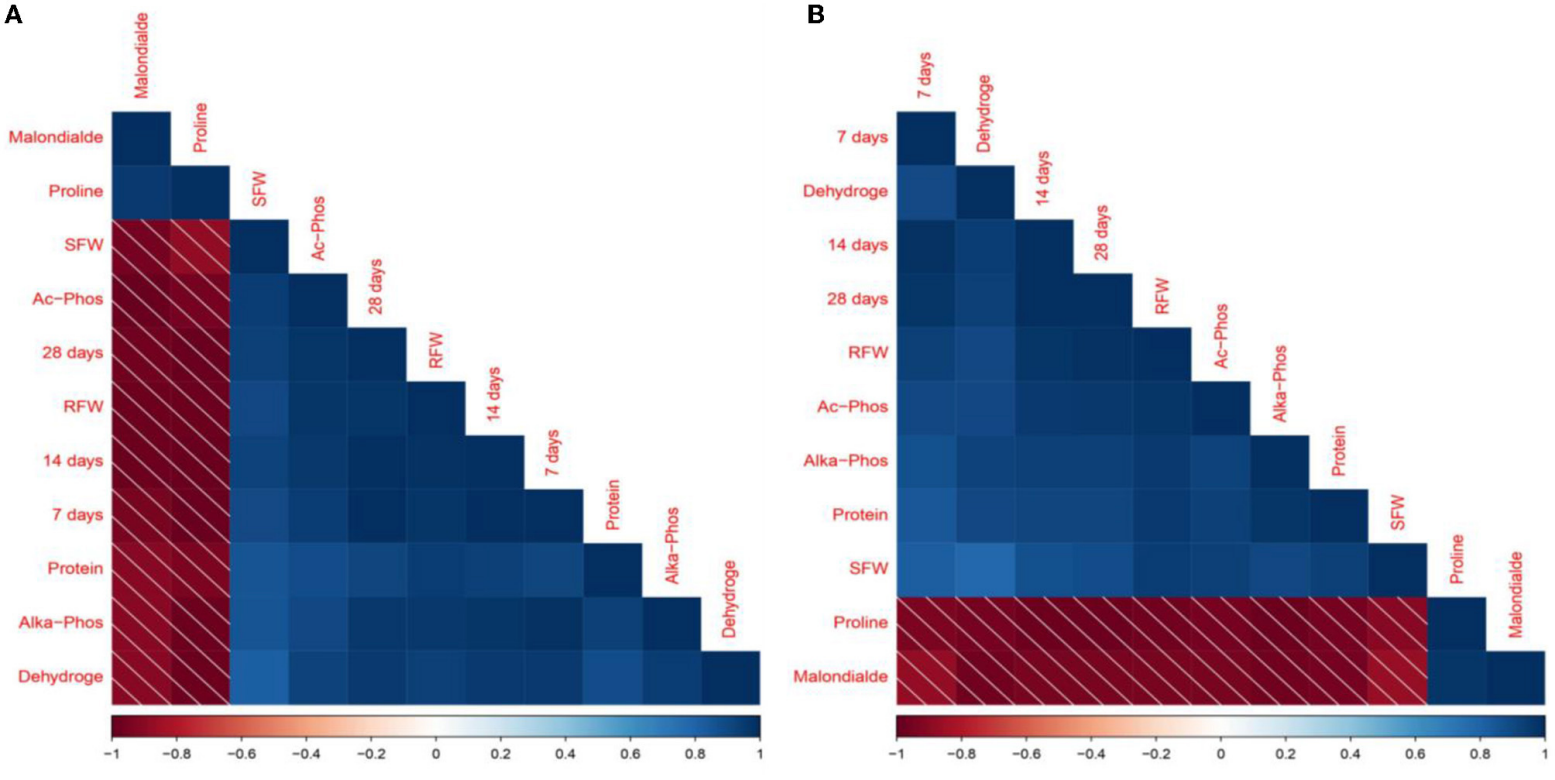
Pearson's correlation among glyphosate breakdown, plant physiological and morphological characteristics, and soil enzymatic activities at 100 **(A)** and 200 **(B)** mg kg ^−1^ glyphosate. 7 days, degradation on 7 days; 14 days, degradation on 14 days; 28 days, degradation on 28 days; SFW, shoot fresh weight; RFW, root fresh weight; Proline, free proline contents; Protein, protein contents; Malonalde, malonaldehyde contents; Dehydroge, dehydrogenase production; Ac-Phos, acid phosphatase; Alka-Phos, alkaline phosphatase.

The Pearson correlation heat map matrix revealed a strong correlation among plant morphological and physiological characteristics, antioxidant activity in plants, and enzymatic activities in the soil, as shown in Figures 6A, B. Glyphosate degradation on 28 days at 100 mg kg^−1^ showed a positive correlation to shoot fresh weight (*r* = 0.93), degradation on 28 days at 100 mg kg^−1^ was highly correlated with root fresh weight (*r* = 0.98), and proline and malonaldehyde were negatively correlated with the degradation on 28 days that was *r* = −0.99 and *r* = −0.96, respectively. Furthermore, acid phosphatase activities positively correlated with shoot and root fresh weight (*r* = 0.94 and *r* = 0.97), respectively, at 100 mg kg^−1^.

### 3.9 Glyphosate-degrading microbes' relation with plant and soil enzymatic activities

**Figure 7:**
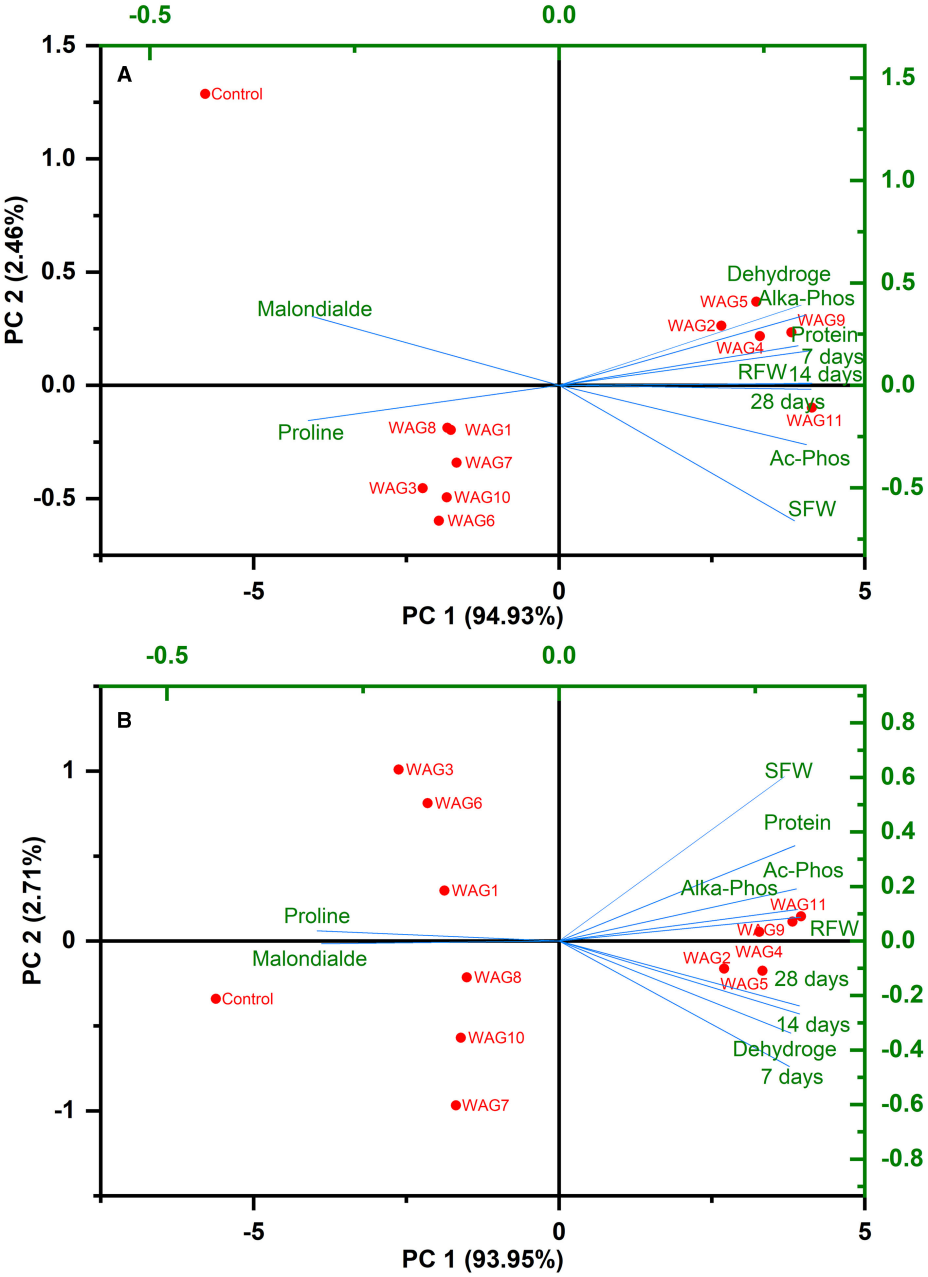
Principal component analysis illustrating the correlations among resistant bacteria, glyphosate degradation, plant morphological and physiological characteristics, and soil enzymatic activities at **(A)** 100 and **(B)** 200 mg kg^−1^ glyphosate.

Most of the characteristics employed in this investigation had positive correlations with the WAG11, WAG9, WAG5, WAG4, and WAG2 strains, as shown by the PCA analysis in Figure 7 at (A) 100 and (B) 200 mg kg^−1^. The key parameters comprise degradation of GP, shoot and root fresh weight, total protein contents, free proline contents, malondialdehyde, acid phosphatase, alkaline phosphatase, and dehydrogenase at 100 mg kg^−1^ and 200 mg kg^−1^. Out of all the bacterial strains tested, WAG11, WAG9, WAG5, WAG4, and WAG2 fared the best when the factors mentioned above were considered.

### 3.10 Bacterial sequencing

**Figure 8:**
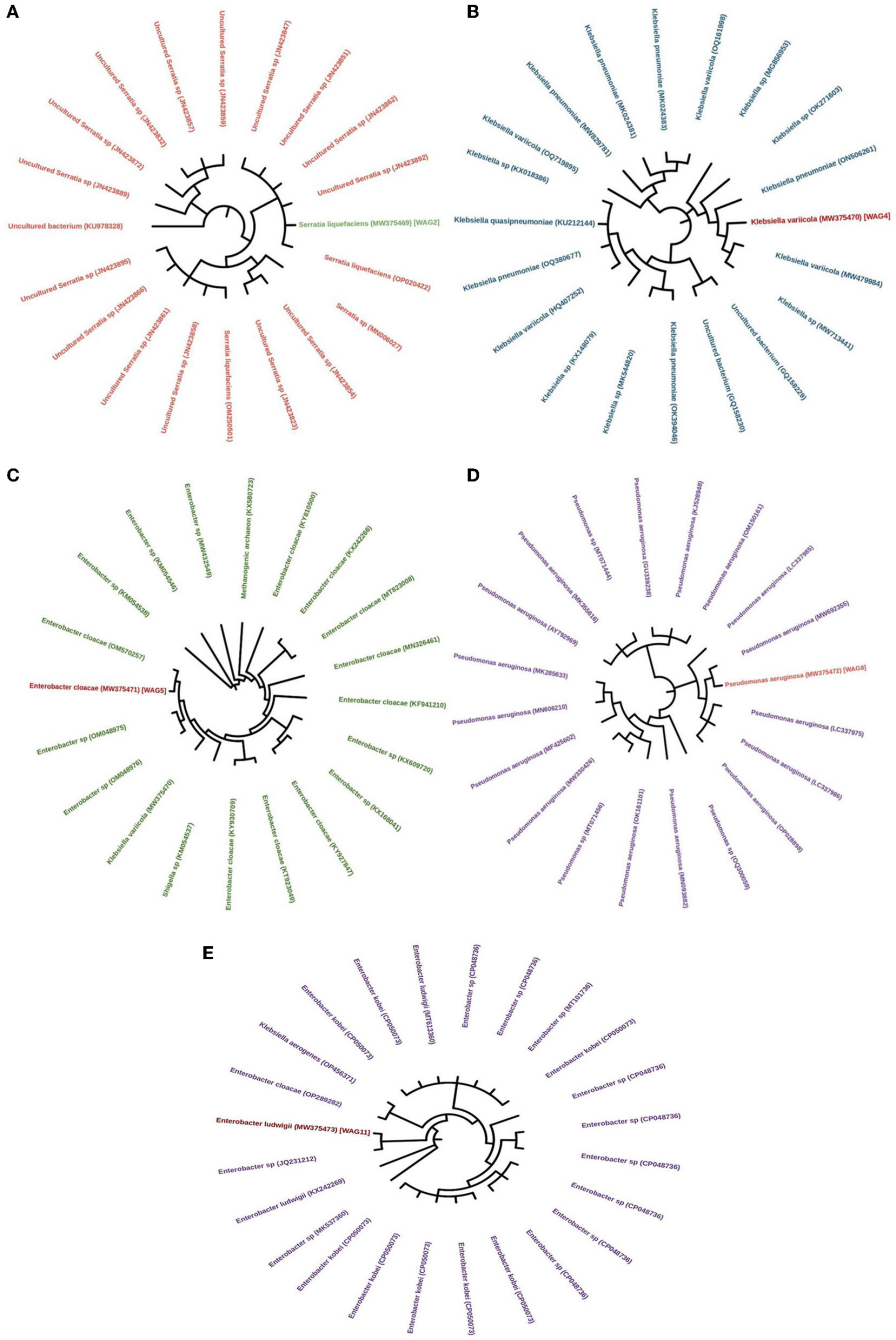
Neighbor-joining tree illustrating the phylogeny of the isolated bacterial strains, **(A)** *Serratia liquefaciens*, **(B)** *Klebsiella variicola*, **(C)** *Enterobacter cloacae*, **(D)** *Pseudomonas aeruginosa*, and **(E)** *Enterobacter ludwigii*. Accession numbers are presented in parentheses.

The evolutionary history was inferred by utilizing the neighbor-joining method, as proposed by Saitou and Nei (1987). The tree that exhibits optimality is depicted. The bootstrap test was conducted with 1000 replicates to determine the percentage of replicate trees in which the corresponding taxa were clustered. The results are displayed below the branches (Felsenstein, 1992). The maximum composite likelihood method was utilized to calculate the evolutionary distances, expressed in the number of base substitutions per site (Tamura et al., 2004). This analysis involved 20 nucleotide sequences in WAG2, WAG4, WAG5, and WAG9, while 24 nucleotide sequences were used in the case of WAG11. The unspecific places in each sequence pair were eliminated (pairwise deletion option). There were a total of 816 (WAG2), 709 (WAG4), 706 (WAG5), 705 (WAG9), and 737 (WAG9) positions in the final dataset (Kumar et al., 2016). 16s rRNA sequencing showed that WAG2 was identified as *Serratia liquefaciens*, WAG4 as *Klebsiella variicola*, WAG5 as *Enterobacter cloacae*, WAG9 as *Pseudomonas aeruginosa*, and WAG11 as *Enterobacter ludwigii*, with a similarity of 95–100% as shown in Figure 8.

## 4 Discussion

Before the start of the study, it was hypothesized that the application of indigenous GP-degrading growth-promoting bacteria could enhance maize growth and degrade GP as well. The GP is widely used in weed management as pest pressures pose significant challenges to crop production. Such as soil pollution and yield losses, i.e., in both peanut (*Arachis hypogaea* L.) and soybean (*Glycine max* L.) fields, weeds were responsible for more than 70% of the total loss in production (Shrestha et al., 2021). Several weeds grow in maize (*Zea mays* L.) crops, but the presence of a single weed, i.e., the growth of maize, may be drastically slowed by *Palmer amaranth*. According to some studies, having 0.5 to 8 *P. amaranth* plants m^−1^ per row may reduce maize output by 11–91% (Karimmojeni et al., 2021). The GP is used to eradicate these weeds, significantly reducing crop yields. Moreover, based on the reports on GP toxicity to ecology and humans and recent reports on the use of GP for weed control, the WHO reclassified GP as a probable human carcinogen in 2015 (Davoren and Schiestl, 2018). So, this study is based on the isolation and use of indigenous isolated microbes for the degradation of GP to improve plant growth, soil enzymatic activities, and soil health to bestow GP tolerance under GP-contaminated soils.

The results of the current study indicated that using GP-based herbicides had different negative impacts on the seed germination index and seedling establishment. Isolated bacterial strains WAG11, WAG9, WAG5, WAG4, and WAG2 on average significantly improved the germination of maize seeds at both 100 and 200 mg kg^−1^ application levels of GP. These findings presented in Table 3 were in line with Gomes et al. (2017) and Helander et al. (2019) as they reported the noxiousness of GP herbicide concentrations (0 to 50 mg L^−1^) reduced respiration rates in *D. wilsonii* seed caused to a lower seed germination index. Mondal et al. (2017) stated that *Pisum sativum* germination of seedlings under control conditions (0 mg L^−1^) was 100% after 3 days of treatment, but at 3 and 4 mg L^−1^ GP, germination was reduced to 55 and 40%, respectively. Seeds contaminated with GP do not accumulate hydrogen peroxide (H_2_O_2_) because of the presence of antioxidants, for instance, ascorbate peroxidase (AP) and catalase production by microbes. Glyphosate and GP-based (Roundup) formulation adversely influenced these enzyme activities coupled with the disturbance in the functioning of the electron transport chain (ETC) in mitochondria with Complex III as its specific site. The same phenomenon also happens when the herbicide GP weakens *D. wilsonii* seed germination by disturbing the mitochondrial ETC, causing decreased energy (ATP) production (Gomes et al., 2017). This could be due to reduced GP stress by producing catalase enzyme produced by isolated bacterial strains, which reduces hydrogen peroxide production in the seed. The oxidoreductase enzyme may ultimately degrade the GP residue by using GP-degrading pathways.

The results relevant to plant growth and physiological attributes revealed that GP concentration above threshold levels above excessive concentrations of GP harmed the plant's morphological and physiological characteristics and disturbed soil enzymatic activities. However, the inoculation of isolated GP-tolerant rhizobacterial strains overcomes the impact's effects on GP. The GP-tolerant inoculation improved the plant's growth promotion characteristics, soil enzymatic activities, and physiological parameters in maize. These improvements might be due to the production of different growth-related enzymes such as IAA, siderophore, and phosphorus-solubilizing bacteria. Moreover, GP toxicity might also be reduced by the production of varying enzymes, i.e., ACC deaminase, oxidase, catalase, and exopolysaccharides.

Furthermore, the current research revealed that the GP degradation of GP-based herbicide efficacies differed in the biotic and uninoculated environments as GP degradation was lower under control (uninoculated) conditions at both concentrations of GP (100 and 200 mg kg^−1^). This might be due to the microorganism in the uninoculated control soil (uninoculated) that could not tolerate GP toxicity at higher concentrations. In contrast, the indigenously isolated 11 inoculated (isolated GP-tolerant bacterial strains) treatments showed higher rates of GP biodegradation at both concentrations. Five strains from 11 GP-tolerant strains outperformed at both levels, while almost 100% degradation efficacy was achieved by these strains within 28 days compared to their respective controls. These results, as shown in Figure 2, aligned with the previously reported results by reports (Zhan et al., 2018; Tang et al., 2019; Feng et al., 2020a). It is essential to highlight that the average biodegradation in all inoculated treatments reached 59.6 mg kg^−1^ after 28 days at a dosage of 200 mg kg^−1^ of GP owing to more severe GP stress on bacterial strains growth due to their lack of adaptability to molecular metabolism (Serafini et al., 2022). Hertel et al. (2021) found that 5-enolpyruvylshikimate-3-phosphate (EPSP) synthase was detected in *Bacillus subtilis*, and this enzyme does not permit alterations that increase GP resistance in bacterial strains. However, EPSP synthase activity may be too low for *B. subtilis* to continue if there is even a single change in the amino acid sequence of this enzyme. Excessive GP toxicity may also diminish microbial biomass in GP-contaminated spiking soil by inducing structural alterations in the protein structure of isolated bacterial strains. Blake and Pallett (2018) revealed that concentrations of 10–100 mg kg^−1^ GP resulted in noticeably less soil microbial biomass. Recently, it has been shown that the administration of GP at the authorized dose (<10 mg kg^−1^) alters the activity of enzymes, microbial functions, and community composition in plant rhizospheres and bulk soil in greenhouse tests (Helander et al., 2019; Baćmaga et al., 2021). Higher GP concentrations also disturb the soil nutrient cycles by disturbing the consequences of GP on earthworms (Owagboriaye et al., 2021), and their relations with symbiotic mycorrhizal fungi have also been informed by Hagner et al. (2019).

The plant growth-promoting parameters, i.e., root and shoot lengths of maize, revealed that the applied GP concentration contamination significantly declined the root and shoot and root lengths due to the reason that GP application changes in the soil nutrients availability, characteristics, and microbial communities by decreasing the microorganism's richness and enhancing the phytopathogenic fungi (Kepler et al., 2020). According to Gomes et al. (2019), glyphosate has been found to decrease root elongation, potentially by disrupting auxin production and impeding cell division. At both concentrations (100 and 200 mg kg^−1^), root and shoot lengths were improved due to the availability of phosphorus and auxin production at the vegetative growth. Moreover, isolated bacterial strains produced GP oxidoreductase enzyme, breaking the carbon–phosphorous bond and making phosphorous and sarcosine available. Glyphosate can also play an essential role in influencing phosphate on plants in soils because GP and phosphorus share the exact adsorption mechanisms and sites in soils. The study's findings presented in Figure 3 were aligned with Gomes et al. (2019). However, it is more possible that the harmful impact of GP is related to its primary mode of action: the reluctance of 5-enolpyruvylshikimate-3-phosphate synthase (EPSPS), an enzyme produced during the shikimate pathway. Indole tryptophan precursors and tryptophan are significant products of the shikimic acid pathway and are utilized in producing indole-3-acetic acid (IAA) (Gomes et al., 2019). Therefore, GP may inhibit the biosynthesis of auxin in plant tissues. It is known that auxin stimulates the growth process in roots (Fu and Harberd, 2003), and IAA is linked to the prior activation of the cell division cycle (Tognetti et al., 2012).

The current study revealed that these isolated bacteria improved maize shoot and root fresh weight at 100 mg kg^−1^ of GP application in soil. This has not yet been reported in earlier ambient studies. The improvements in the root and shoot weights were because biodegradation of GP not only de-contaminates the soil but also improves soil health and nutrient availability. These microorganisms likely improve plant growth via the solubilization of nutrients (insoluble) in the soil and the production of vital plant hormones. Glyphosate-degrading bacteria can also produce indole acetic acid (IAA), siderophore, and phosphorous-by-phosphorous solubilizing bacteria that can help to increase plant growth through plant available phosphorus supply (Kumar et al., 2018). Correspondingly, the stress generated in the occurrence of reactive oxygen species and reactive nitrogen species has also been examined to change the morphology of plant roots by a decline in growth. Isolated bacterial strains showed positive results by producing ACC deaminase under GP stress (Shahid and Khan, 2018). It was also demonstrated that inoculating maize with *Pseudomonas* and *Enterobacter* increased the plant's lateral root production (Rai et al., 2020). The growth of adjacent roots in cucumber (*Cucumis sativus* L.) was increased by *Bacillus spp*. inoculation (Gouda et al., 2018). Improved biodegradation of GP and restoration of soil biotic factors may be achieved with the use of plant growth-promoting rhizobacteria.

Evaluation of relative water content in plants is an effective method to illustrate the water content in plants. A decrease in the relative water content (RWC) is the earliest significant effect of GP stress. Consequently, GP stress depressingly influenced the RWC in maize leaves (Zobiole et al., 2011). Under GP stress, plants suffer osmotic stress, which indicates decreased water uptake. Additionally, unnecessary abscisic acid (ABA) production under GP stress stimulates stomata's stomatal closure, indicating no or less water uptake through roots, meaning low relative water contents (Sofo et al., 2004). Microbial assistance showed improved RWC in plants, which has already been revealed by Naseem and Bano (2014). The improvement in the RWC under GP-resistant bacterial strains inoculation may be due to bacterial-induced improvement in the root length that can facilitate the plant's uptake of more water under the GP-stressed condition (Al-Shwaiman et al., 2022).

It was also observed that the minimum reduction of proline contents in inoculated treatment was by WAG11 and MDA at both concentrations. The findings of this study, as shown in Figure 4, align with those reported by Sergiev et al. (2006), who noted that the enhanced proline production could disrupt osmoregulation in elevated GP concentration in control (uninoculated) soil. The highest production of MDA contents might be due to the uptake of GP. The GP treatment usually lessened the growth and pigment content in maize. It enlarged the bio-membrane lipid peroxidation (MDA), the flux of ions in leaf tissue, and the activity of peroxidase and catalase. This could be because all these assume that along with the reserve of its specific target site, the GP action indicates the chain-radical reactions (oxidative stress). Even though the direct mechanism by which GP stimulates the generation of reactive oxygen species is mysterious, based on current findings, it could be believed that this is a secondary effect of the blocked shikimate pathway. Inoculation of GP-tolerant rhizobacterial strains initiated a considerable decline in the production of both free proline and MDA. The findings of the current study presented in Figure 4 are in line with the findings of Shahid and Khan (2018), who reported that it was due to the inoculation of isolated bacteria because the negative impacts of GP on plant physiology and growth were considerably reduced by producing different stress copping enzymes such as ACC deaminase, catalase, oxidase, and exopolysaccharides.

The study has also revealed that GP toxicity negatively affected the soil enzymatic activities in control (uninoculated) soil. This decrease may be due to an indirect decline of microbial activities under GP-contaminated spiked soil, suggesting that the soil enzyme production by native microbes may be reduced under 100 and 200 mg kg^−1^ GP concentration. However, a remarkable increase in all the assessed soil enzymatic activities such as acid phosphatase, alkaline phosphatase, and dehydrogenase was recorded and examined in this study, which is presented in Table 4 in line with the findings of García-Orenes et al. (2010) and Meena et al. (2020), who reported a substantial increase of enzymatic activities in the GP-contaminated inoculated soil. Better dehydrogenase activities in this experiment were attended by considerable support in soil nutrient profile and plant growth. Soil microorganisms excrete various enzymes (cellulases, ureases, dehydrogenases, phosphatases, etc.), which can utilize GP as a substrate. Notably, the soil exhibiting natural cover demonstrated the highest phosphatase activity levels. According to Smith et al. (2011), this particular enzyme is predominantly synthesized by plant roots and is commonly linked to mycorrhiza and other fungi.

**Figure 9:**
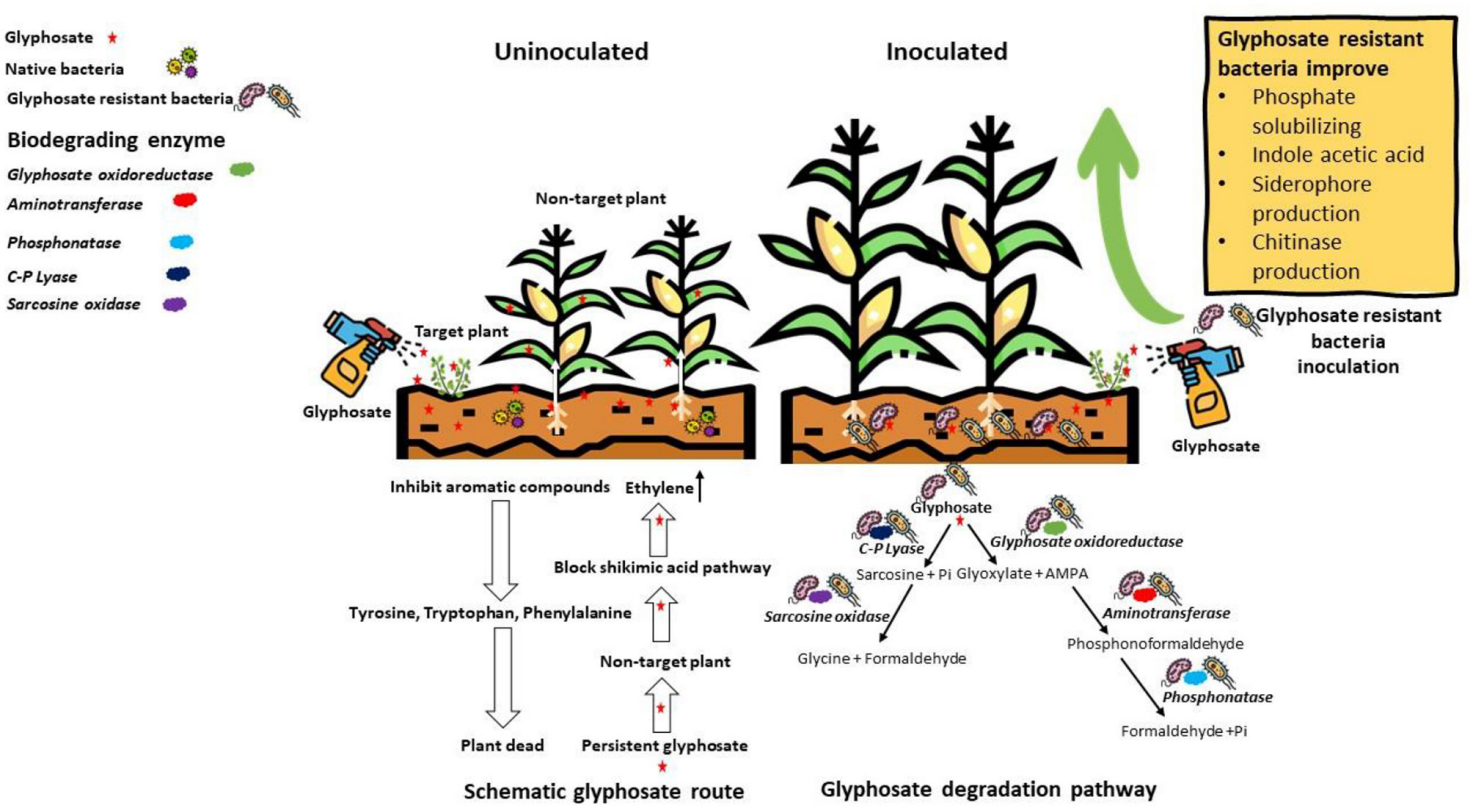
Illustration of schematic glyphosate route and its degradation pathways.

In this study, the biodegradation of GP was efficiently done by 5 out of 11 isolated bacterial strains. These five strains were WAG2, WAG4, WAG5, WAG9, and WAG11. It has been reported that two different pathways can degrade GP. The initial step of GP degradation is the catalytic action of GP oxidoreductase, which produces the AMPA and glyoxylate (Singh et al., 2020b), as shown in Figure 9. *Pseudomonas sp*. biodegrades the GP via AMPA and glycine pathway, and it was revealed that it utilizes glyoxylate and formaldehyde for its growth (La Cecilia and Maggi, 2018). Additionally, bacterial strain *A. atrocyaneus* ATCC 13,752 breaks down GP into AMPA and then completely converts it into CO_2_, but the CO_2_ acquired is not the end product of AMPA (Sidhu et al., 2019). The AMPA, an intermediate compound, gets released into the environment or further biodegrades due to another enzyme (Sun et al., 2019). Usually, AMPA behaves as a substrate for carbon phosphorous cleavage enzyme, which produces Pi and methylamine. *Arthrobacter* sp. GLP-1, *A. atrocyaneus* ATCC 13752, and *Pseudomonas* sp. LBr utilized it as a source of phosphorous (La Cecilia and Maggi, 2018). Lately, *O. anthropi* GPK3 isolates demonstrated the new AMPA degradation pathway, broken into phosphonic–formaldehyde via transaminase enzyme and then further to formaldehyde via transaminase enzyme phosphatase. Another GP degradation pathway involves carbon phosphorous lyase and produces Pi and sarcosine, for instance, *Pseudomonas* sp. PG2982 utilized GP via carbon phosphorous lyase to generate sarcosine, which further gets broken down to formaldehyde and glycine by sarcosine oxidase (Feng et al., 2020b), as shown in Figure 9. The bacterial strain *Arthrobacter* sp. utilizes glycine for the biosynthesis of protein. This fate of GP metabolites has been evaluated with the assistance of isotope tagging. Isolated GP-tolerant bacterial strains (WAG11, WAG9, WAG5, WAG4, and WAG2) may use any of these pathways to degrade GP residue from the soil.

Considering the capacity for GP degradation among these five bacteria, it is evident that *Enterobacter ludwigii* has a notable potency compared to the other strains. Furthermore, it has the potential to be practiced in many agricultural applications. The solubilization of P, N fixation, and production of plant hormones have the potential to enhance plant growth, yield, and stress tolerance. Hence, it is plausible to consider *Enterobacter ludwigii* as a promising candidate for practice as a bio-fertilizer and bio-remediator in the context of promoting sustainable agricultural practices. Further investigation is necessary to have a comprehensive understanding of the molecular mechanisms and genetic determinants of this phenomenon under varying environmental circumstances.

## 5 Conclusion

The study conducted under ambient conditions revealed that five out of the eleven isolated GP-tolerant bacterial strains demonstrated significant efficacy in degrading GP, enhancing plant growth, reducing oxidative stress, and improving soil health (enzymatic activities) in GP-spiked soil at a concentration of 100 mg kg^−1^, as compared to 200 mg kg^−1^. As a result, the herbicide residue is decreasing, and its toxicity to maize plants is reduced. The bacterial strains that exhibited promising results were WAG2, WAG4, WAG45, WAG9, and WAG11. Once the bacterial strains are submitted to culture-collecting facilities, they will be made available to other researchers for access. One potential strategy for agricultural applications involves the introduction of bacterial strains capable of thriving in soil contaminated with GP by breaking down the GP and enhancing plant development. Our plan consists of testing isolated bacterial candidate strains in their natural environment, specifically field soils, to evaluate their impact on different crop plants.

## Data availability statement

The raw data supporting the conclusions of this article will be made available by the authors, without undue reservation.

## Author contributions

WM-U-D: Conceptualization, Formal analysis, Methodology, Writing – original draft. FC: Data curation, Investigation, Methodology, Writing – original draft. SB: Investigation, Project administration, Supervision, Writing – original draft. MA: Conceptualization, Data curation, Validation, Writing – review & editing. HA: Conceptualization, Data curation, Investigation, Validation, Visualization, Writing – review & editing. ZF: Data curation, Software, Writing – original draft. UZ: Investigation, Resources, Visualization, Writing – review & editing. FH: Formal analysis, Validation, Writing – review & editing. AA: Conceptualization, Validation, Visualization, Writing – review & editing. MA: Funding acquisition, Writing – review & editing.
